# Supplementary material for: Subfunctionalisation and self-repression of duplicated E1 homologues finetunes soybean flowering and adaptation
Source: Nat Commun. 2024 Jul 23;15:6184. doi: 10.1038/s41467-024-50623-3 (PMC11263555; doi:10.1038/s41467-024-50623-3)
Supplement: Supplementary file 1 — Supplementary Information [file 41467_2024_50623_MOESM1_ESM.pdf]

Subfunctionalisation and self-repression of duplicated *E1* homologues finetunes soybean flowering and adaptation

Fang *et al.*

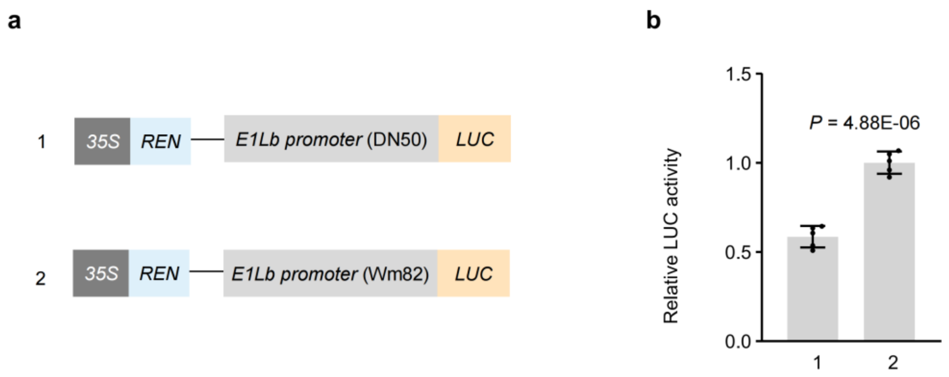

Supplementary Fig. 1. Mutations alter *E1Lb* promoter activity.
(**a**) Schematic of constructs used for the transient assay. (**b**) Relative LUC activity from the *E1Lb* promoters. Data are the mean ± S.D. of n = 5 biological replicates. A two-sided Student's *t*-test was used to generate the *P* values. Source data are provided as a Source Data file.

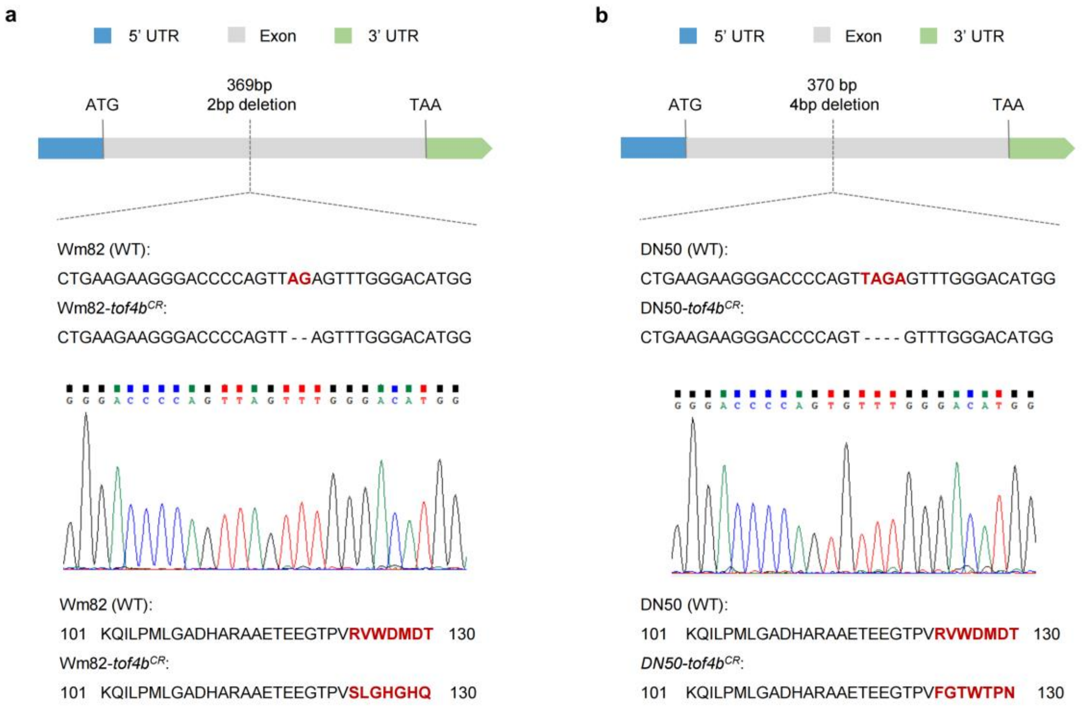

Supplementary Fig. 2. ***E1Lb*** **CRISPR–Cas9 mutagenesis strategy.**
(**a**) Top: Schematic of the *E1Lb* and the location of the edits induced by CRISPR–Cas9 gene editing. In the Wm82 background, an edited line with 2-bp deletion results in a frameshift at the 124th codon (out of 193). Middle: Sanger sequencing chromatogram of the edited line showing the sequence around the mutation. Bottom: Amino-acid alignment of E1Lb from wild-type Wm82 and the edited line Wm82-*tof4b*^{CR} with changes to the reading frame. (**b**) Top: Schematic of the *E1Lb* and the location of the edits induced by CRISPR–Cas9 gene editing. In the DN50 background, an edited line with a 4-bp deletion results in a frameshift at the 124th codon (out of 193). Middle: Sanger sequencing chromatogram of the edited line showing the sequence around the mutation. Bottom: Amino-acid alignment of E1Lb from wild-type DN50 and the edited line DN50-*tof4b*^{CR} with changes to the reading frame.

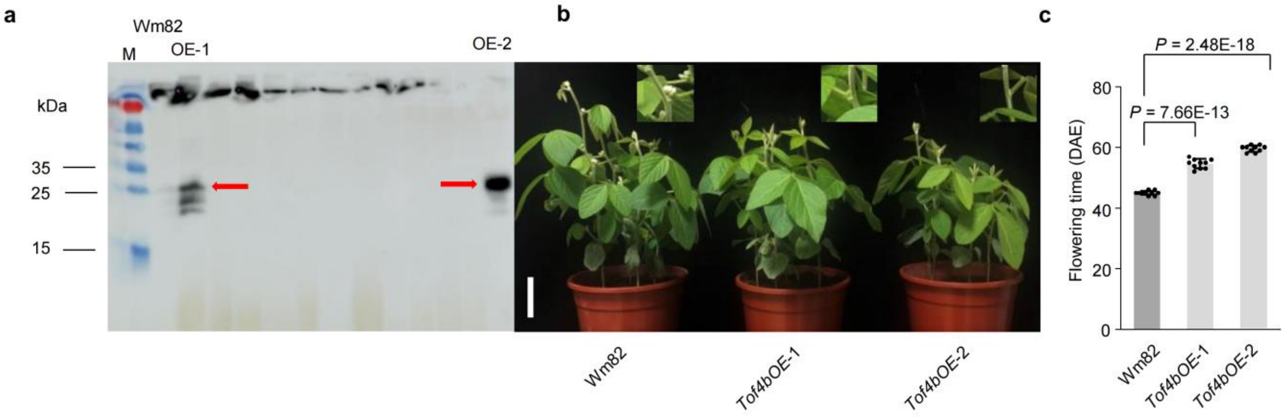

Supplementary Fig. 3. Phenotypes of Wm82 and *Tof4-OE* lines under long-day photoperiod.
(**a**) Immunoblot analysis of wild-type Wm82 and two genetically independent stable-transgenic *Tof4b-OE* lines (OE-1, 2) expressing E1Lb–3xFLAG probed with anti-FLAG antibodies. Samples were harvested at ZT 4 from 20-d old plants grown under long-day conditions. The red arrow indicates the Tof4b–3xFLAG target (expected size = 23.2 kDa). M: Molecular-weight marker. (**b**) Phenotypes of Wm82 and two stable transgenic *Tof4b*-OE lines grown under long-day conditions in a growth chamber. Scale bar = 10 cm. (**c**) Flowering time of Wm82 and two genetically independent transgenic *Tof4b-OE* lines. Data are the mean ± S.D. of n = 10 plants. The two-sided Student's *t*-test was used to generate the *P* values. Source data are provided as a Source Data file.

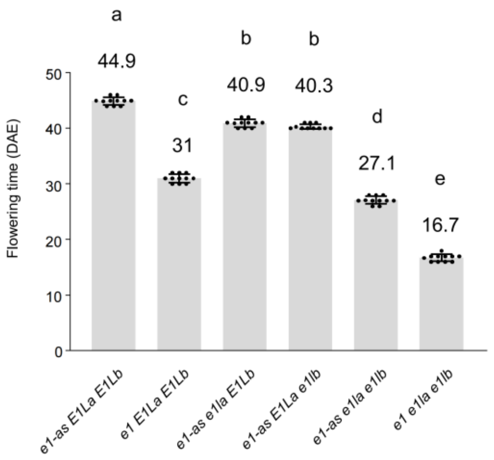

Supplementary Fig. 4. The effects of *E1*, *E1La* and *E1Lb* on soybean flowering time.
Flowering time of Wm82 (*e1-as E1La E1Lb,* containing a partial loss-of-function *e1* allele designated 'as' for amino-acid substitution), *e1 E1La E1Lb* (carrying a null *e1* allele), *e1-as e1la E1Lb*, *e1-as E1La e1lb*, *e1-as e1la e1lb*, and *e1 e1la e1lb* mutants grown under long-day conditions. Data are the mean ± S.D. of n = 10 plants and the mean values for each genotype are listed above each bar. Different letters indicate statistically significant differences as determined by one-way ANOVA ($P < 0.05$). DAE, days after emergence. Source data are provided as a Source Data file.

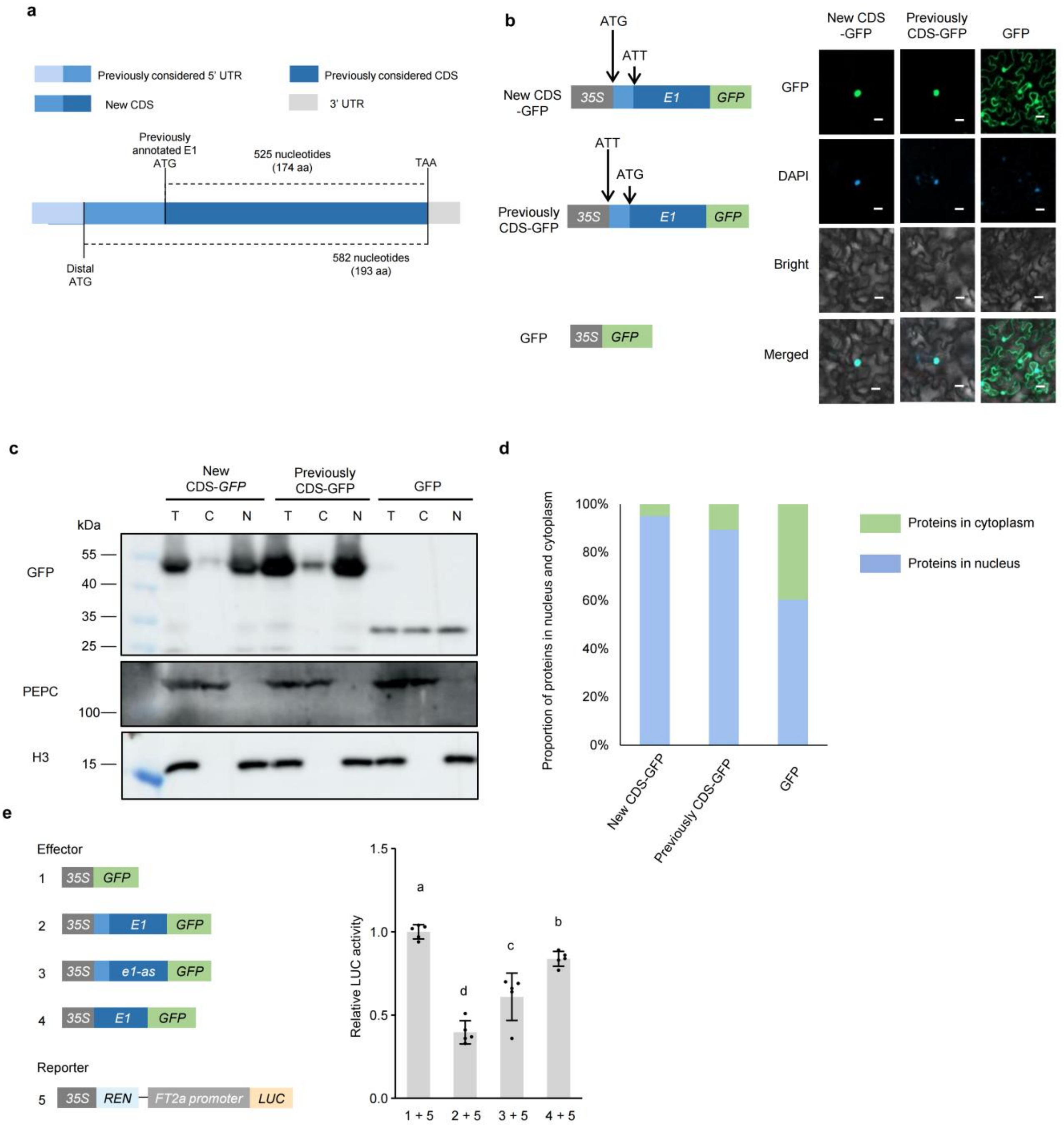

Supplementary Fig. 5. Confirmation of the E1 translation-initiation site.
(**a**) Schematic of the c.v. Wm82-E1 *E1* coding sequence and UTRs. (**b**) Left: Schematic of constructs used for subcellular localisation of E1 isoforms with different translation-initiation sites in transiently transgenic *N. benthamiana* leaves. GFP was fused to C-termini and constructs were under the control of the *CaMV35S* promoter. ATT: Mutation inserted to replace ATG. Right: Confocal microscopy of the constructs shown on the left. Scale bars = 25 μm. Three independent biological replicates were performed. (**c**) Subcellular-fractionation analysis of various proteins as summarised in (**b**). *N. benthamiana* leaves were left to transiently express for 48–72 h after infiltration the indicated constructs before harvest and protein extraction. E1–GFP isoforms were detected using anti-GFP antibodies, PEPC was detected with anti-PEPC antibodies as the cytoplasmic-fraction marker and H3 proteins were detected using anti-H3 antibodies as the nuclear-fraction marker. T: Total, C: Cytoplasmic fraction, N: Nuclear fraction. Three independent biological replicates were performed. (**d**) Proportion of proteins in nucleus and cytoplasm. All immunoblot bands in (**c**) were analysed for band grayscale values using ImageJ to estimate relative protein intensity. (**e**) Transcriptional-inhibitory activity of E1–GFP proteins on the *FT2a* promoter region with different translation-initiation site. Data are the mean ± S.D. of n = 5 biological replicates. Different letters indicate statistically significant differences determined by one-way ANOVA ($P < 0.05$). Source data are provided as a Source Data file.

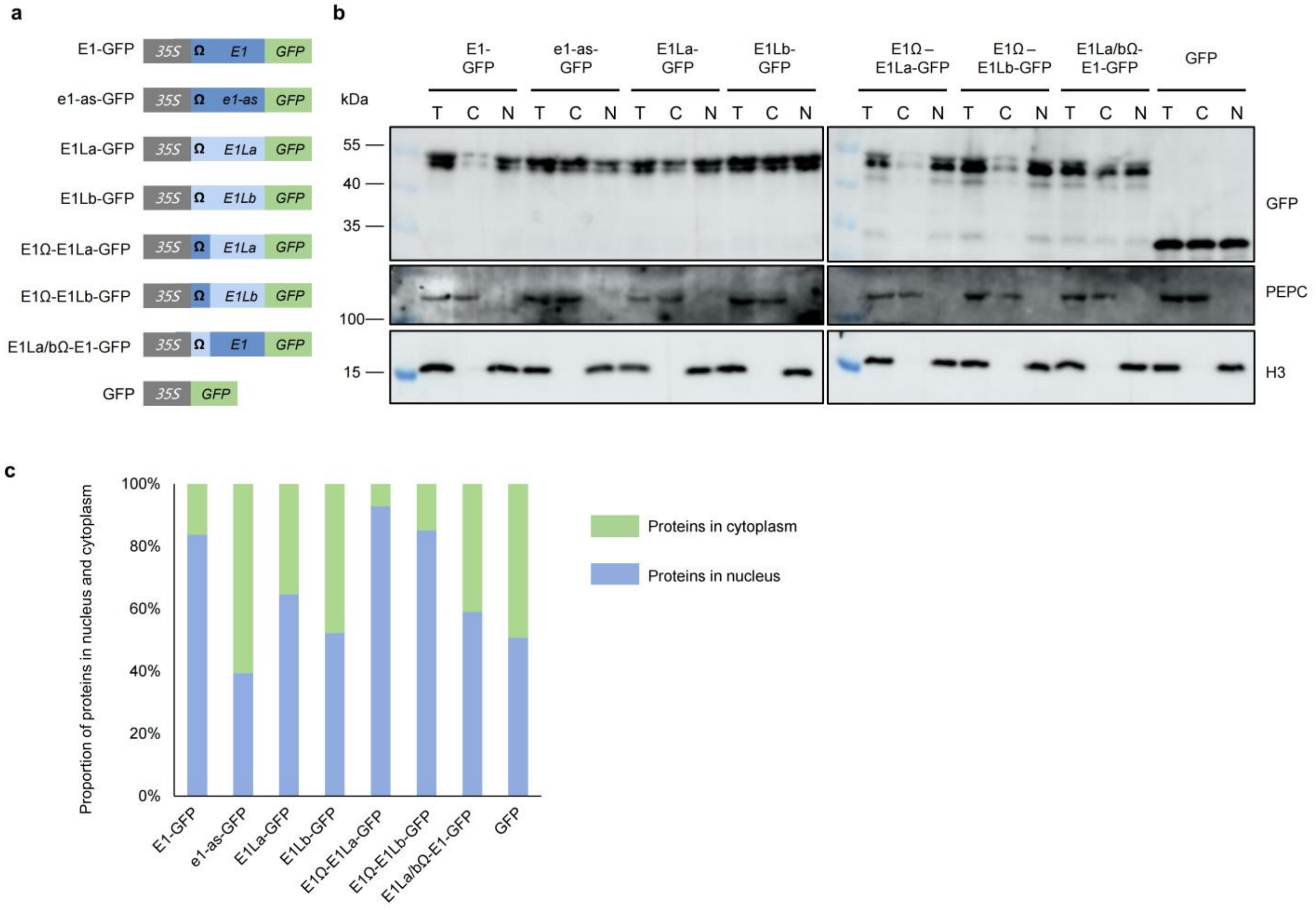

Supplementary Fig. 6. Subfunctionalisation of soybean E1 proteins.
(**a**) Schematic of the constructs used for subcellular localisation of soybean E1 isoforms in transiently transgenic *N. benthamiana* leaves. GFP was fused to C-termini and constructs were under the control of the *CaMV35S* promoter. 'Ω' depicts the N-terminus peptide. (**b**) Subcellular-fractionation analysis of E1-family proteins as summarized in (**a**). *N. benthamiana* leaves were left to transiently express for 48–72 h after infiltration the indicated constructs before harvest and protein extraction. E1–GFP isoforms were detected using anti-GFP antibodies, PEPC was detected with anti-PEPC antibodies as the cytoplasmic-fraction marker and H3 proteins were detected using anti-H3 antibodies as the nuclear-fraction marker. T: Total, C: Cytoplasmic fraction, N: Nuclear fraction. Three independent biological replicates were performed. (**c**) Proportion of proteins in the nucleus and cytoplasm. All immunoblot bands in (**b**) were analysed for band grayscale values using ImageJ to estimate relative protein intensity. Three independent biological replicates were performed. Source data are provided as a Source Data file.

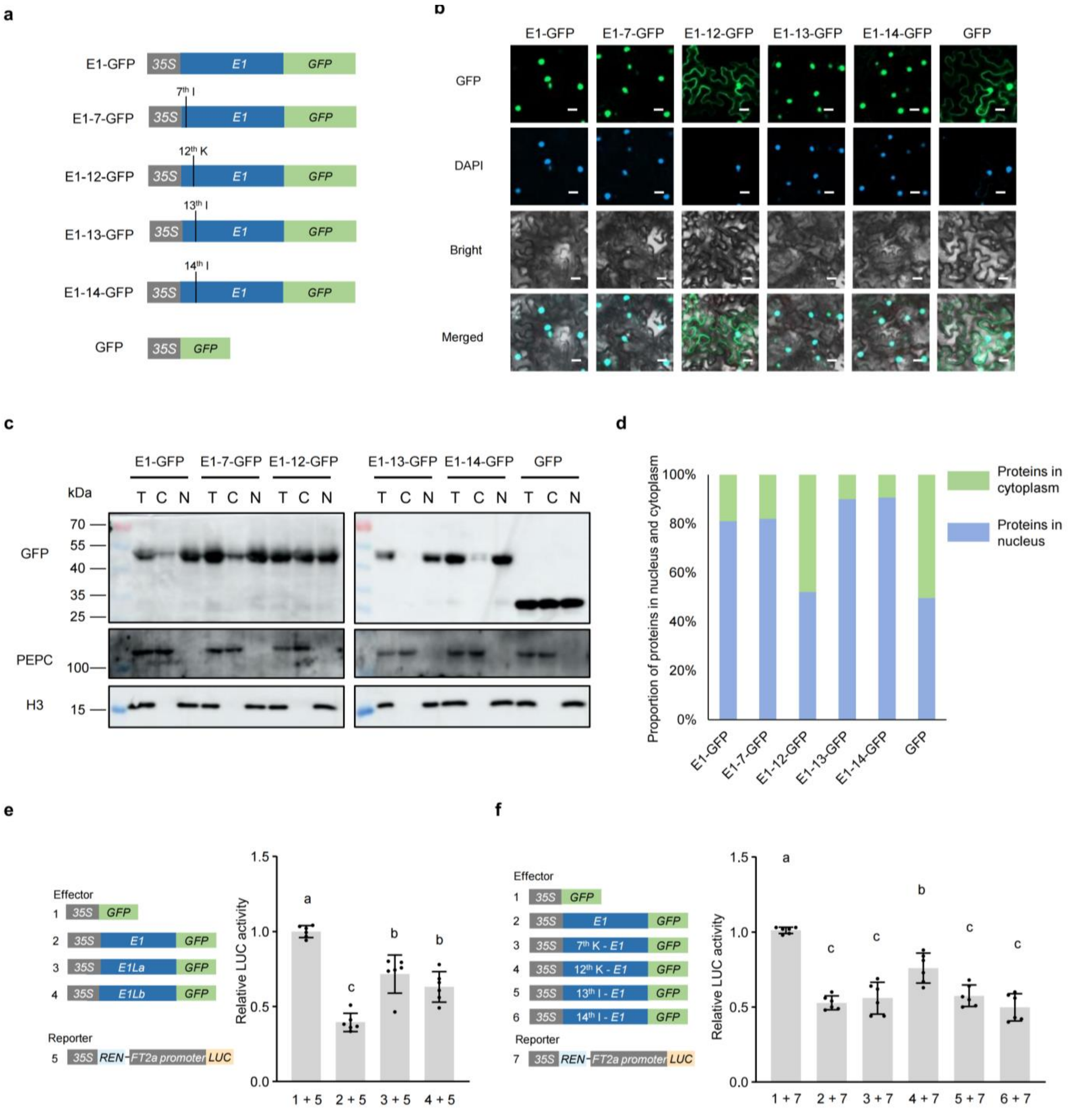

Supplementary Fig. 7. Amino-acid substitutions affect E1 nuclear localisation.
(**a**) Schematic of constructs used for subcellular localisation of soybean E1 isoforms with different amino-acid substitutions (position 7 S→**I**, position 12 T→K, position 13 T→**I**, and position 14 L→**I**) in transiently transgenic *N. benthamiana* leaves. GFP was fused to C-termini and constructs were under the control of the *CaMV35S* promoter. (**b**) Confocal-microscopy analysis of subcellular localisation of soybean E1 isoforms as summarised in (**a**). Scale bar = 25 μm. Three independent biological replicates were performed. (**c**) Subcellular-fractionation analysis of various proteins as summarized in (**a**). *N. benthamiana* leaves were left to transiently express for 48–72 h after infiltration the indicated constructs before harvest and protein extraction. E1–GFP isoforms were detected using anti-GFP antibodies, PEPC was detected with anti-PEPC antibodies as the cytoplasmic-fraction marker and H3 proteins were detected using anti-H3 antibodies as the nuclear-fraction marker. T: Total, C: Cytoplasmic fraction, N: Nuclear fraction. Three independent biological replicates were performed. (**d**) Proportion of proteins in the nucleus and cytoplasm. All immunoblot bands in (e) were analysed for band grayscale values using ImageJ to estimate relative protein intensity. (**e**) Transient dual-reporter assay of transcriptional-inhibitory activity of E1, E1La and E1Lb. Left: Constructs used for transient assay. Right: Relative LUC activity for the indicated construct pairs. Data are the mean ± S.D. of n = 6 biological replicates. Different letters indicate statistically significant differences determined by one-way ANOVA ($P < 0.05$). (**f**) Transient dual-reporter assay of transcriptional-inhibitory activity of E1 proteins with different amino-acid substitutions. Left: Constructs used for transient assay. Right: Relative LUC

activity for the indicated construct pairs. Data are the mean ± S.D. of n = 6 biological replicates. Different letters indicate statistically significant differences determined by one-way ANOVA ($P < 0.05$). Source data are provided as a Source Data file.

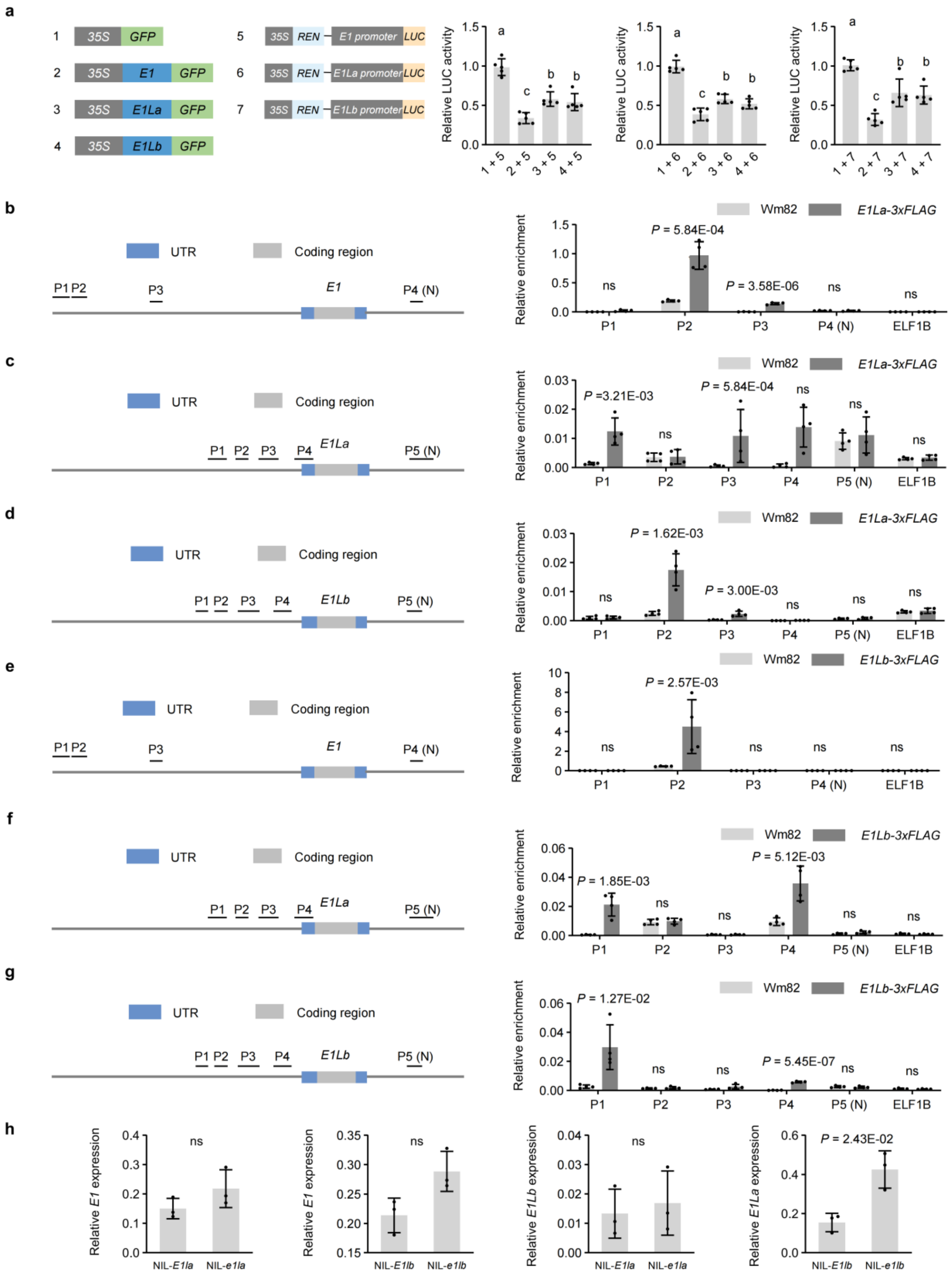

Supplementary Fig. 8. *E1*, *E1La* and *E1Lb* all exhibit self-repression capability and the ability to suppress their homologous genes. (**a**) Transcriptional inhibitory activity of three E1 proteins to their promoters. Different letters indicate statistically significant differences determined by one-way ANOVA ($P < 0.05$). (**b-g**) ChIP–qPCR assay for enrichment of E1La–Flag and E1Lb-Flag at the promoter of *E1*, *E1La* and *E1Lb*. ELF1b served as a negative control. Data are the mean ± S.D. of n = 4 biological replicates. The two-sided Student's *t*-test was used to generate the *P* values. ns indicate no significant difference ($P > 0.05$). ns indicate no significant difference ($P > 0.05$). (**h**) Expression levels of *E1*, *E1La* and *E1Lb* in the NILs of *E1Lb* and *E1La*. Data are the mean ± S.D. of n = 3 biological replicates. The two-sided Student's *t*-test was used to generate the *P* values. ns indicate no significant difference ($P > 0.05$). ns indicate no significant difference ($P > 0.05$). Source data are provided as a Source Data file.

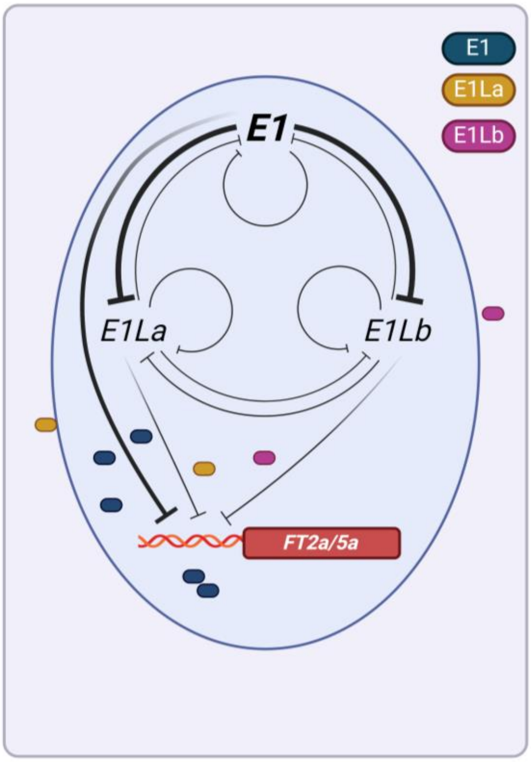

Supplementary Fig. 9. Model of *E1* family-member functions in the soybean photoperiodic flowering pathway.
E1, *E1La* and *E1Lb* all possess the ability for self-repression and repression of their homologous genes. Among these repressive effects, the inhibition of *E1La* and *E1Lb* by *E1* predominates. Mutations in the N-termini of E1La and E1Lb affect their nuclear-localisation capacity, with E1 maintaining full activity and E1La and E1Lb having only partial activity. Subsequently, the three E1 homologues inhibit the expression of *FT2a* and *FT5a*, and consequently flowering time, in a hierarchical manner. The ellipsis represents the nucleus. Supplementary Fig. 9 Created with BioRender.com (https://biorender.com/) released under a Creative Commons Attribution-NonCommercial-NoDerivs 4.0 International license.

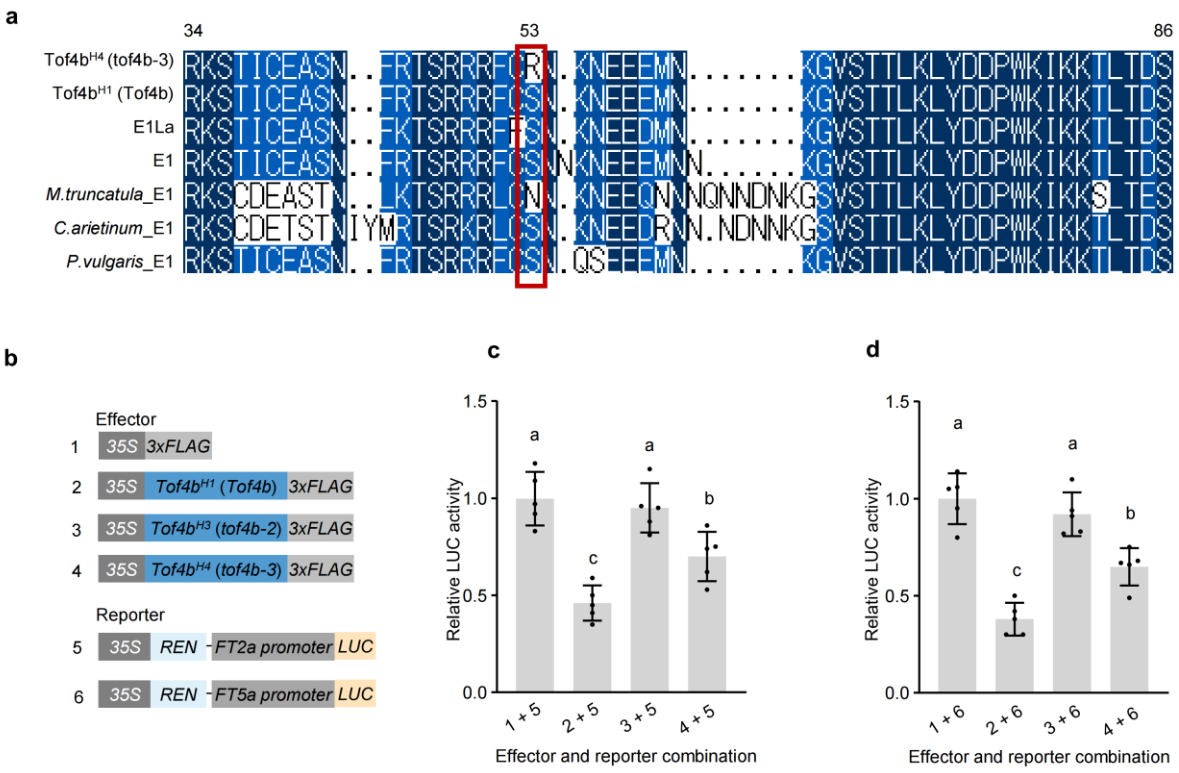

Supplementary Fig. 10. Identification of a functional mutant *tof4b*.
(**a**) Alignment of Tof4b sequences from each haplotype and its orthologues in *M. truncatula*, *C. arietinum* and *P. vulgaris* around the S53R mutation in cultivated soybean. The numbers indicate residue numbering. (**b**) Schematic of the constructs used for the transient report-gene assay. (**c, d**) Relative LUC activity driven by the *FT2a* (**c**) and *FT5a* promoters (**d**) under the regulation Tof4b effector alleles shown in (**b**). Data are the mean ± S.D. of n = 5 biological replicates and different letters indicate statistically significant differences determined by one-way ANOVA ($P < 0.05$). Source data are provided as a Source Data file.

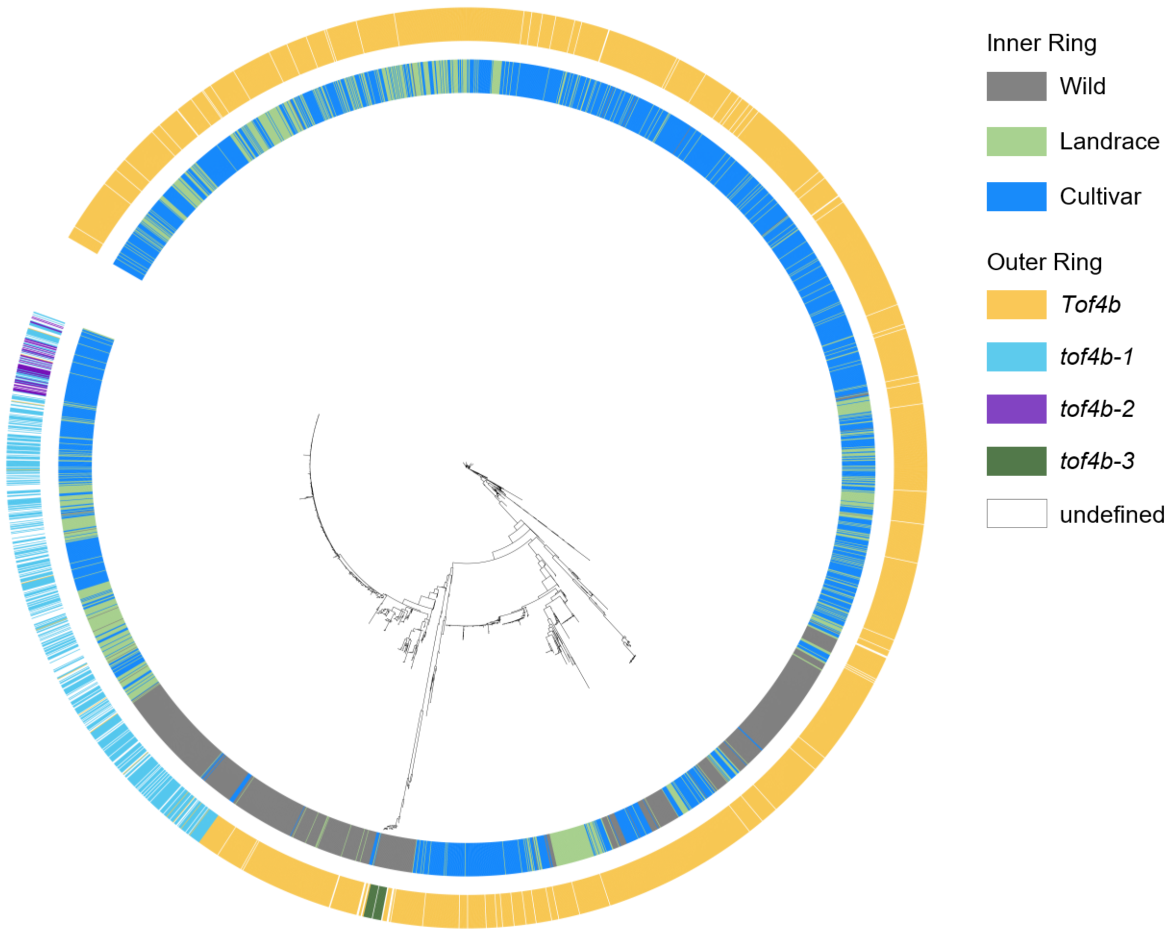

Supplementary Fig. 11. Phylogenetic tree constructed with re-sequencing data around the *Tof4b* locus. Phylogenetic tree constructed with re-sequencing data around the *Tof4b* locus (2 Mb) from 3050 wild and cultivated soybeans sourced from northern China and the *Tof4b* haplotypes of each accession. 'Undefined' indicates that there is missing re-sequencing data in the *Tof4b* gene region for this accession, therefore unable to genotype *Tof4b*.

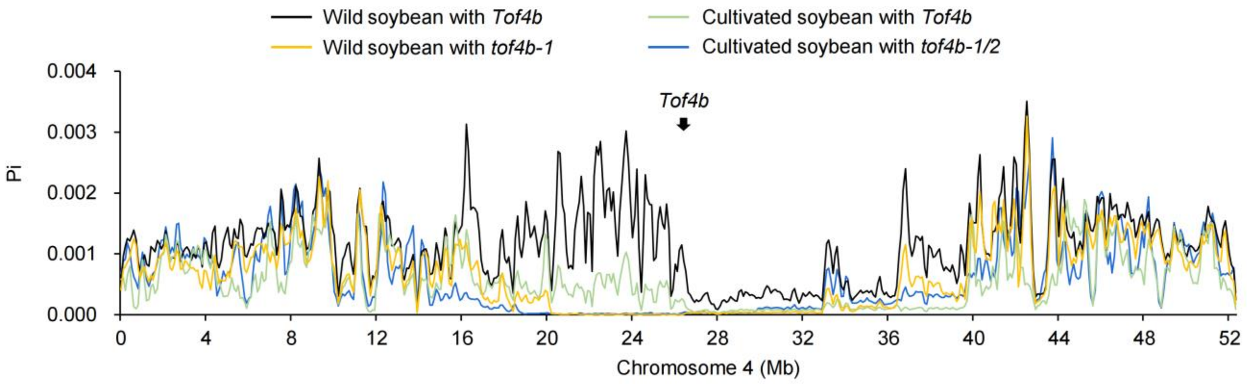

Supplementary Fig. 12. Pi values on chromosome 4 for wild and cultivated soybeans.
Pi values within chromosome 4 for wild soybean with *Tof4b,* wild soybean with *tof4b-1*, cultivated soybean with *Tof4b,* and cultivated soybean with *tof4b-1/2.*

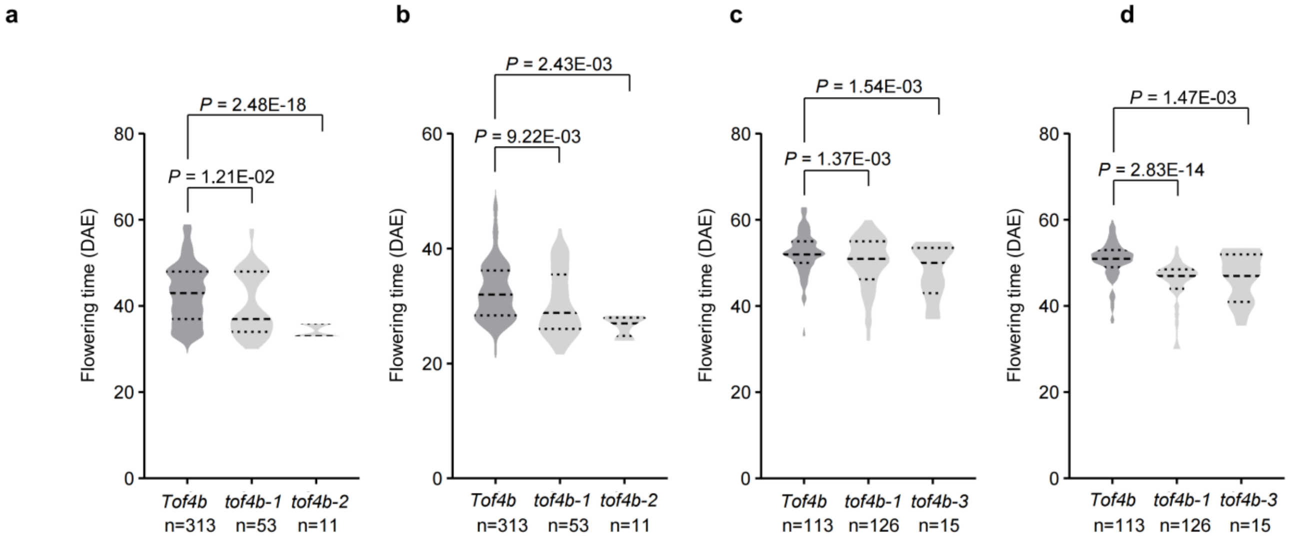

Supplementary Fig. 13. Flowering time of field-grown soybeans in Harbin. Flowering time of cultivated soybeans in *e1 e2* backgrounds in 2020 (**a**) and 2021 (**b**) and for wild soybeans in 2020 (**c**) and 2021 (**d**) under no special selection on backgrounds. Data are the mean ± S.D. The two-sided Student's *t*-test was used to generate the *P* values. DAE, days after emergence. n represents the number of accessions of each genotype. Source data are provided as a Source Data file.

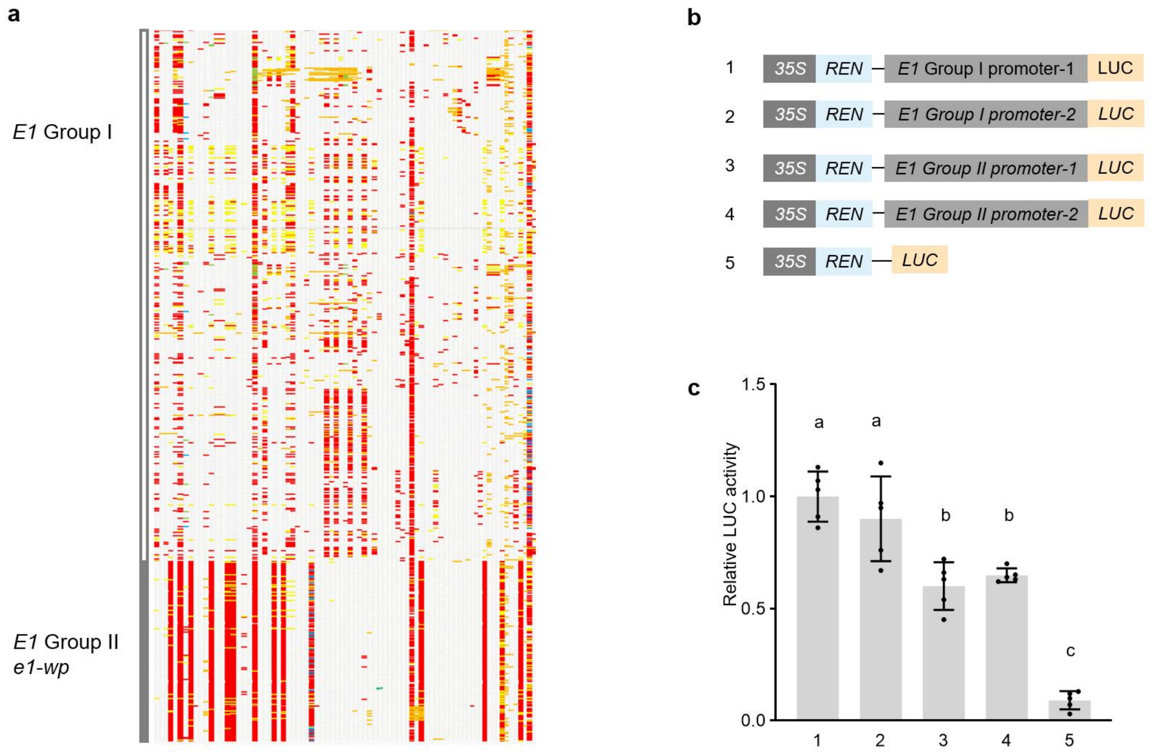

Supplementary Fig. 14. Mutations in the *E1* promoters of wild soybeans from high latitudes fall into two groups with altered activity.
(**a**) Haplotypes in the *E1* promoter region (3 kb) of wild soybeans from high latitudes. The heat map shows genotypes at each nucleotide position. Red cells represent SNPs or InDels based on comparison to the Wm82 reference genome. Yellow cells represent sites with low quality. Six SNPs and 4 InDels in the *E1* promoter segregate the population into two subgroups. (**b**) Schematic of the constructs used for transient reporter-gene assay. (**c**) Relative LUC activity driven *E1* promoter groups as summarised in (**b**). Data are the mean ± S.D. of n = 5 biological replicates and different letters indicate statistically significant differences by one-way ANOVA ($P < 0.05$). Source data are provided as a Source Data file.

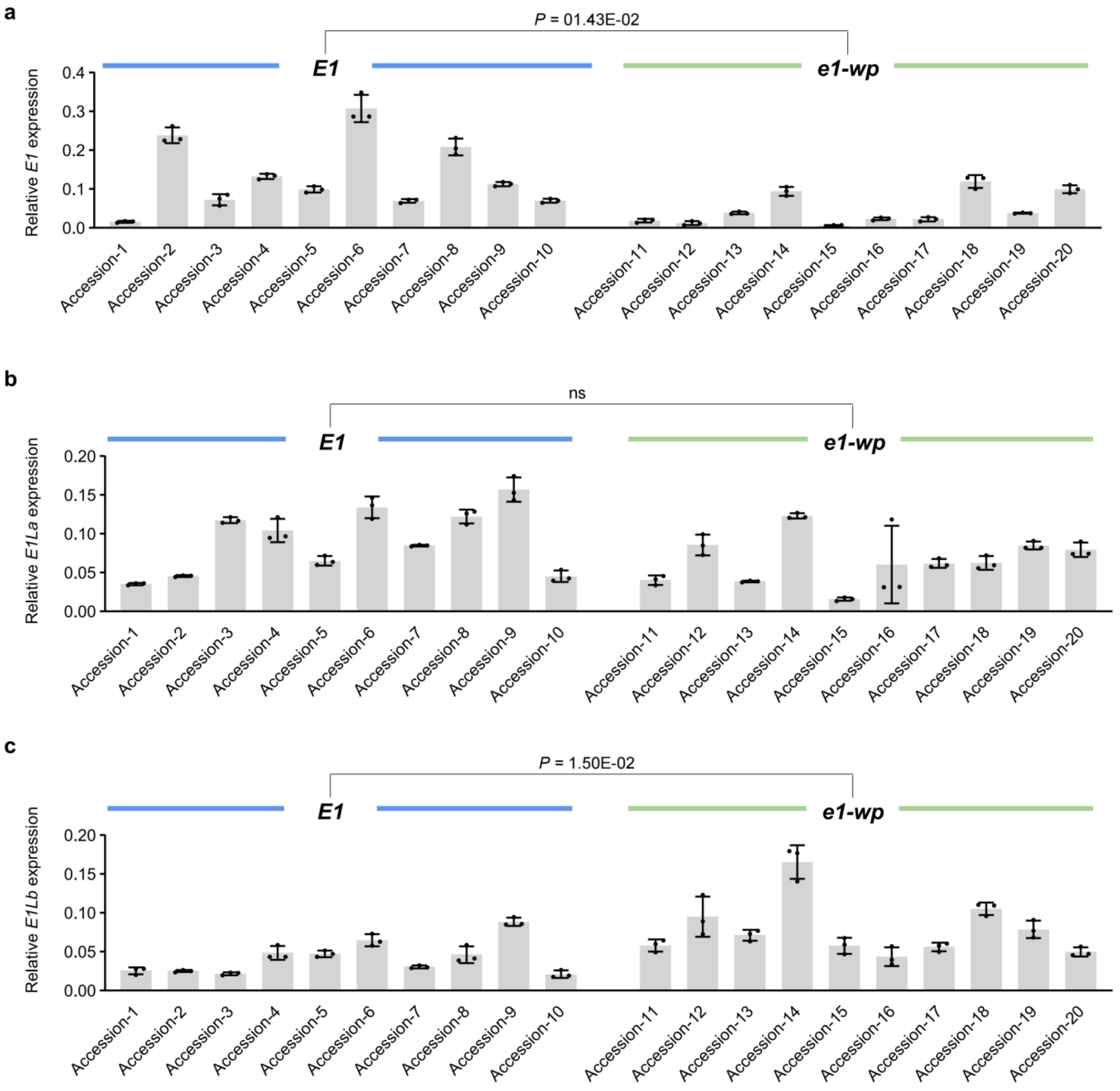

Supplementary Fig. 15. Expression levels of *E1*, *E1La*, and *E1Lb* in wild soybeans with *E1* or *e1-wp* alleles. Expression level of *E1* (**a**), *E1La* (**b**) and *E1Lb* (**c**) in ten wild soybean accessions carrying *E1* alleles and ten accessions carrying *e1-wp* alleles. Accessions are listed in Supplementary Table. 6. Data are the mean ± S.D. of n = 3 biological replicates for every accessions. The two-sided Student's *t*-test was used to generate the *P* values. ns indicate no significant difference ($P > 0.05$). List of the accessions was provided in Supplementary Table 1. Source data are provided as a Source Data file.

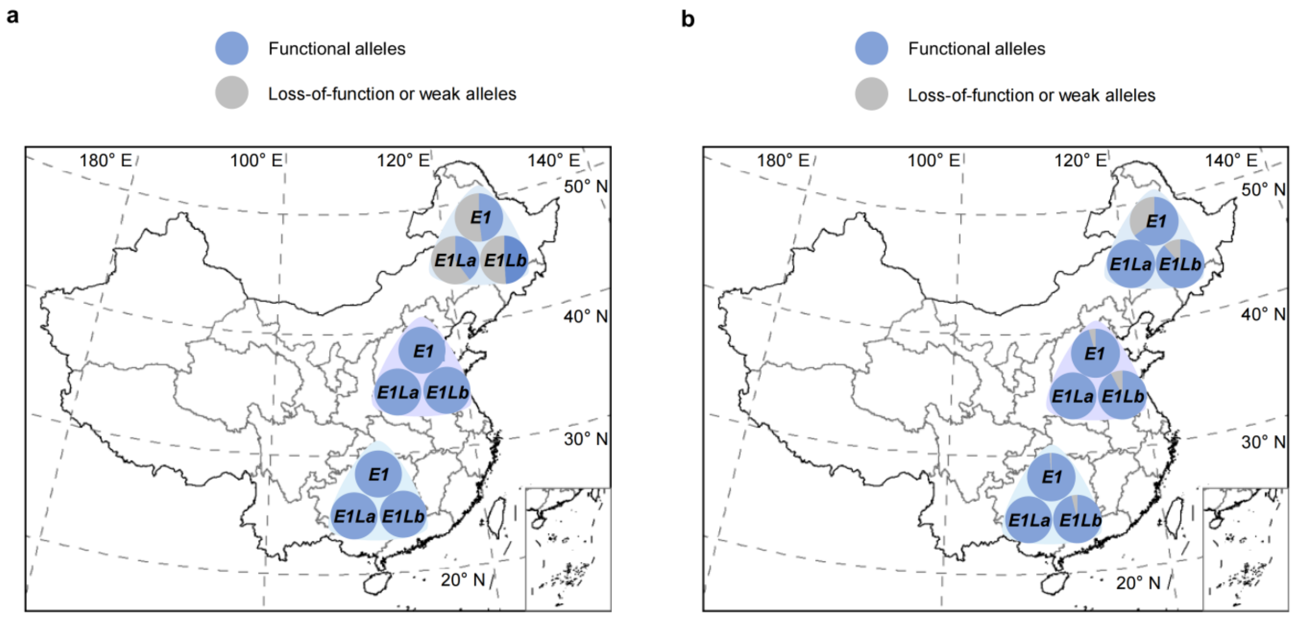

Supplementary Fig. 16. Geographical distribution of *E1* genes in China.
Loss-of-function or weak alleles of *E1*, *E1La* and *E1Lb* are highly enriched in high-latitude regions. Data from a panel of 851 wild soybeans (**a**) and a panel of 1,083 cultivated soybeans (**b**) are shown. Gray panels represent loss-of-function or weak-allele carriers and blue panels represent functional-allele carriers. The map was drawn using ArcGIS v.10.3 software for desktop (https://desktop.arcgis.com/en/).

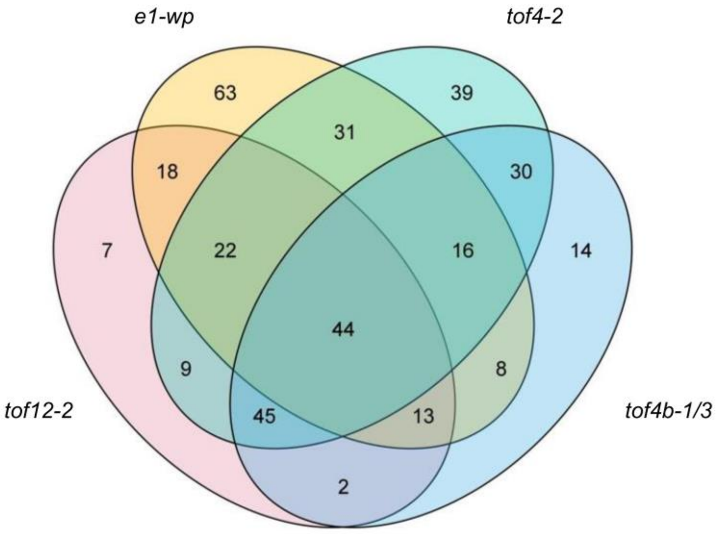

Supplementary Fig. 17. Relationships amongst wild soybean accessions from high-latitude regions that carry major mutant alleles at the *E1*, *Tof4*, *Tof4b* and *Tof12* loci.

Venn diagram showing the number of wild soybean accessions from high-latitude regions that carry the major mutant alleles at the *E1*, *Tof4*, *Tof4b* and *Tof12* loci, and their relationships. The numbers at the intersecting regions of the ellipses represent the count of accessions that exhibit two or more mutant-allele variations simultaneously. The population used for this analysis consists of wild soybeans from northern China and Russia.

Supplementary Table 1. Accessions used in Supplementary Fig. 15.

Individual	Name of accession
Accession-1	GDW029
Accession-2	GDW103
Accession-3	GDW008
Accession-4	GDW043
Accession-5	GDW061
Accession-6	GDW052
Accession-7	ZKW0067
Accession-8	GDW095
Accession-9	WR_19
Accession-10	ZKW0067
Accession-11	WR_30
Accession-12	WR_30
Accession-13	ZKW0102
Accession-14	ZKW0007
Accession-15	ZKW0007
Accession-16	ZKW0058
Accession-17	ZKW0287
Accession-18	ZKW0121
Accession-19	ZKW0076
Accession-20	ZKW0272
