# Supplementary material for: Subfunctionalisation and self-repression of duplicated E1 homologues finetunes soybean flowering and adaptation
Source: Nat Commun. 2024 Jul 23;15:6184. doi: 10.1038/s41467-024-50623-3 (PMC11263555; doi:10.1038/s41467-024-50623-3)
Supplement: Supplementary file 3 — Description of Additional Supplementary Files [file 41467_2024_50623_MOESM3_ESM.pdf]

File Name: Supplementary Data 1
Description: Primers in this study.

File Name: Supplementary Data 2
Description: Accessions in Fig 4c and their *Tof4b* allele.

File Name: Supplementary Data 3
Description:Accessions in Fig 4d and their *Tof4b* allele.

File Name: Supplementary Data 4
Description: *Tof4b* alleles of all accessions in this work.

File Name: Supplementary Data 5
Description: Alleles of *Tof4*, *Tof4b*, *E1*, *Tof12*, and *Tof5* of wild soybeans in high latitudes.
